# Supplementary material for: Patterns of Intron Gain and Loss in Fungi
Source: PLoS Biol. 2004 Nov 30;2(12):e422. doi: 10.1371/journal.pbio.0020422 (PMC532390; doi:10.1371/journal.pbio.0020422)
Supplement: Table S1 — Also available at http://genes.mit.edu/NielsenEtAl/. (4.3 MB ZIP). [file pbio.0020422.st001.zip › NielsenEtAl/html/500.html]

AN2014.1.NCU01455.1.MG06904.1.FG01900.1


```
 CLUSTAL W (1.82) Multiple Sequence Alignments - Introns Inserted


Sequence 1: NCU01455.1	682 aa
Sequence 2: MG06904.1	671 aa
Sequence 3: FG01900.1	643 aa
Sequence 4: AN2014.1	902 aa
Alignment Length: 943 aa
Number Identitical Residues: 177 aa
Alignment Score (without introns) 9930


MG06904.1 	-----~------------------------------------------------~-----
NCU01455.1	-----~------------------------------------------------~-----
FG01900.1 	-----~------------------------------------------------~-----
AN2014.1  	MQVGG2WAGAARQILNPEFNLPLGADPSFYVISRLGSATWPTPASRTGVKSSAV1KPQPQ
          	 . ..  :.:: .  ... . . .:..:    :  .::: .:.:: :. .:::  .....

MG06904.1 	---------------------------------------~--------------------
NCU01455.1	---------------------------------------~--------------------
FG01900.1 	---------------------------------------~--------------------
AN2014.1  	NHSRTRTSCSVDDAYEAILVGGGWLRPTIPWRLVGAAEL0AIFPYHSILSKRQKVTCSSA
          	. : : ::.: ..: .:   ...   .: .    .::.  :  .  :  :. .. :.:::

MG06904.1 	------------------------------------------------------------
NCU01455.1	------------------------------------------------------------
FG01900.1 	------------------------------------------------------------
AN2014.1  	GFHLRKAIPSPLGSINGDGKAKRVPPRVGSETQTGYSLCTEHGGEVSLESSEYSLQHTGH
          	.    .: .:. .: .....:.  ..  .:.:.:. : .:. ... : .::. : . :. 

MG06904.1 	----------------------~-------------------------MADP-~------
NCU01455.1	----------------------~-------------------------MSDS-~------
FG01900.1 	----------------------~-------------------------MPQD-~------
AN2014.1  	SCSAQKWCCSPAHWSLCFTYGD2NQVTCKVRQRLATNCAGSKLGSGTHLPDAQ0CPVSGK
          	:.::.. ..:.:  : . : .. .. :..  .  ::..:.:. .:.: :.: . .. :..

MG06904.1 	-------------------AVSALNSLLRGTEIDDHDEVLRLANAAIRSSSSPSSPEFRT
NCU01455.1	-------------------AIAALNSLLRGTSIDDHEEALKLANSAIKAAKG-NPADLTT
FG01900.1 	-------------------PAAALSALLQQSSIEDHEEVLKAANAALKANKT-----DTD
AN2014.1  	HSSLLGQLRPHRETHEIAMSTQSLSSLLSRASIDDHEEVLRSCDAALAKSRS-----DIQ
          	 ::  ..  .  .: . : .  :*.:**  :.*:**:*.*: .::*:             

MG06904.1 	AQQAKVVALVKLDRYDDALRTIADGGDRLATDCALENAYSLYKTGQLDAAAQVCTNFNTG
NCU01455.1	AQHTRVVALVKLDRFDDALRAIAEGGAALDKTCVMEKAYALYKTGELEAAEQLLETTG--
FG01900.1 	SQHTRIIALLKLDRFEDALRAIADGTPTLHSRIALEHTYALYKTGKLDEATLVLKAAG--
AN2014.1  	AQHVKVVALLKLDRYEDCLRVFEEGGDILKKKAALEYGYTLYKTGHPETAIDVVSSLA--
          	:*:.:::**:****::*.**.: :*   * .  .:*  *:*****. : *  :       

MG06904.1 	HSSSMRGARHVAAQV~AYRAEKFAEAARSYQELLETVAREGQPGEEGELRINMLAAAAQL
NCU01455.1	--INTRGMKHLAAQV~AYRAEKFDKAAELYQEMAENVE-DAMYGEENDLRINILATYSQL
FG01900.1 	-LENKRNLQHVAAQV~AYRAERFDEACNIYSRLLDTDP----ADEENDISINLRATLAQS
AN2014.1  	---GDRGARHLEAQA0TYRAEKFRRTAEIYQGLSRESA--PTVNEENDLRINSWATDAQL
          	   . *. :*: **. :****:* .:.. *. :          .**.:: **  *: :* 

MG06904.1 	EWAGNGHQVAESERQTSRRDMETFETAFNAACGCMARGDYGKAEVLLKRSRA~LCEASEE
NCU01455.1	EMQGKGWAVSEEHKKLSRDELEVFETAYNAACGSIAQGDFAKAAFLFKRARD~LCEASED
FG01900.1 	SWLG--YSVTD---KVAVQDSDGFELCYNAACAHIANGSLETAADLLQRASR~LCDASDD
AN2014.1  	QWKG--HPESVRHTRPTRDDLEAFETVYNAACLSIAKGEFDQAGLLLKRAKE1LCRTSED
          	.  *     :    : :  : : **  :****  :*.*.   *  *::*:   ** :*::

MG06904.1 	LSEEEKQAELIPILVQASYVLTRLGKNDAAIECQSSISISD2ISDESAKFVAQNNSLVLN
NCU01455.1	LSEEDKKAELAPIIVQQAYVATKLGNLEEAARFHEEVVLSD2ISDASTRAIAQSNALILK
FG01900.1 	LTEEDKQAEMRPILAQQAYVFAKLGKLKEALDLYNSLSSIK2EEDPDLALILGNNLVALE
AN2014.1  	LTPEDRDAELLPIAVQQLYVLICQGKSAEAESILEEISIKD2ISELSTKRIGQNNAAIAR
          	*: *::.**: ** .*  **    *:   *    ..:   .  .: .   :  .*    .

MG06904.1 	-HGENPYMTARKAETLSIPTGNSKPFECQASILRRNKQVIELQCQKFRGVRSRTAFALKR
NCU01455.1	-SESNPYMAQRLADTLPVVTGNDRLFEEQATIFRRNKYIIELQAQKFAGVKSKTTKILSK
FG01900.1 	PKSENPYLVERRFSTLTARKSHAKLFHHQSEILLRNRLVVDLQVLKHDGVKRRTGAIIAQ
AN2014.1  	GTTANPYALYEALHEVPDSTDNDKLFDYQYNVMTGNLHAVDLLVHKYDGIIRSTSKALSQ
          	    ***   .    :.  ..: : *. *  ::  *   ::*   *. *:   *   : :

MG06904.1 	ESPDAPSISPAATNLGVLHAAAVSHLQTGKEALKSILPLLESRPDDIGLLLTIIQLYIQT
NCU01455.1	EA--EPTISSSKCDLGALNAAAVSELQTGKTALRRILPLLETRPTDVGLLLTVIQLYIQL
FG01900.1 	TP--QPTTSASLGIVSVLNAAASSQGATGKQLLKNLRGLAQTRPHDIGLALTIVQIQLKE
AN2014.1  	AP--FPSTEASTNLLSVYNVAARAKGETGTPALKAILSAVERRPKDIGLVLTAVQLYVNQ
          	 .   *: ..:   :.. :.** :.  **.  *: :    : ** *:** ** :*: :: 

MG06904.1 	ANPQPGLALLEALFRRLESATS-QDHSDVRFAPGLVALAVALYRLQGRRAAIRAELARAA
NCU01455.1	QNPAQALNLLEAFFKRLEAATASENNADVRFAPGLVALAIALYRQQGRHSAVRSELAKAA
FG01900.1 	GGIGSALSVLESFLQRLEKAGN-VEALNARFSPGLVALTVTLLRVQGRESSAKAELVKAA
AN2014.1  	GNTTSAIVILERTLQLLDESIS-EQDKEIRFNPGLLSVLIALYKREGRKTQIKAELAKAA
          	 .   .: :**  :: *: :    :  : ** ***::: ::* : :**.:  ::**.:**

MG06904.1 	TYWQHQTTREAGSATSLLRHAGMELLHSQDEEDLATAGDAFALLCKQQ-RDRLAEAGLVA
NCU01455.1	SHWQSKQ-KAASSGDSLLREAGIELLRSTHPSDLSTAGEAFTHLVAAQPDDRTATAGLIA
FG01900.1 	THWQHRQ---ASPASSLLEEAGVELMKSSDGKDLLLAGSSFQKLIDEQKGSDIATAGLVA
AN2014.1  	TFWSGQA----AQPTSLLRAAGASLLHSSLASDTSQAAELFKSLYEKTPDDLFAVAGYVA
          	:.*. :     .   ***. ** .*::*   .*   *.. *  *      .  * ** :*

MG06904.1 	SFATSDRSRTQPYLDNLTPVDKLTEGVDISALIDAGVA---VLTPTPVAGKKRS--ADG-
NCU01455.1	SFATSDFAKVQPYLGALSPVEKLTAGVDVSALLDAGVASLPVVTHQQQQGKKRAREDDGE
FG01900.1 	AFATSEPSRVEKHLQNLPPVDSLIEGIDVASLLSAGVA---VSASKPGSSTLKRAAPSG-
AN2014.1  	SHAAIDYSQVEAQAKYLPSVDDLISDIDVAALETAGVAPSSSTTAAAAAAIAGARKRPAP
          	:.*: : ::.:     *..*:.*  .:*:::*  ****. .  :     .        . 

MG06904.1 	-----LAATQEPAKKQRRSRAKLPKDYDPDKQPDPERWLPLRDRSTYRPKGKKGKKRVAD
NCU01455.1	TTGEQQKQQQQAAKKQRRKR-KLPKNYDPAKQPDPERWLPLRDRSSYRPKGRKGKKRAQE
FG01900.1 	---------NEGKSQKRRRKIRLPKNYVEGTKPDPERWLPLRDRSSYRPKGKKGKKKAID
AN2014.1  	---------TKDRVAKRVRKSRLPKDYDPSKTADPERWLPLRDRSTYRPRGRKGKQRAAE
          	          :    :*  : :***:*   . .************:***:*:***::. :

MG06904.1 	STQGGMVKEEGEMLELVGGAGAVKVEKVP--------QGGAGNKKKKKGKK
NCU01455.1	ATQGGYVKGDEETLELAGGAGSLKVEKANPAGSGGGGGGGGGGKKKKKGKK
FG01900.1 	STQGGMVK--EETLELVGGGG-VKVEKAP-----------QASKKKKKGKK
AN2014.1  	RTQGGVVSEKSEETTLSGQQQQQKPQTGG-----------SSQKKKKKGKR
          	 **** *. . *   * *     * :.              . *******:
```
